# New thin-film surface electrode array enables brain mapping with high spatial acuity in rodents

**DOI:** 10.1038/s41598-018-22051-z

**Published:** 2018-02-28

**Authors:** W. S. Konerding, U. P. Froriep, A. Kral, P. Baumhoff

**Affiliations:** 1 Institute of AudioNeuroTechnology and Department of Experimental Otology, ENT Clinics, Stadtfelddamm 34, Hannover Medical School, 30625 Hannover, Germany; 2 Translational Biomedical Engineering, Fraunhofer Institute for Toxicology and Experimental Medicine (ITEM), Nikolai-Fuchs-Strasse 1, 30625 Hannover, Germany

## Abstract

In neuroscience, single-shank penetrating multi-electrode arrays are standard for sequentially sampling several cortical sites with high spatial and temporal resolution, with the disadvantage of neuronal damage. Non-penetrating surface grids used in electrocorticography (ECoG) permit simultaneous recording of multiple cortical sites, with limited spatial resolution, due to distance to neuronal tissue, large contact size and high impedances. Here we compared new thin-film parylene C ECoG grids, covering the guinea pig primary auditory cortex, with simultaneous recordings from penetrating electrode array (PEAs), inserted through openings in the grid material. ECoG grid local field potentials (LFP) showed higher response thresholds and amplitudes compared to PEAs. They enabled, however, fast and reliable tonotopic mapping of the auditory cortex (place-frequency slope: 0.7 mm/octave), with tuning widths similar to PEAs. The ECoG signal correlated best with supragranular layers, exponentially decreasing with cortical depth. The grids also enabled recording of multi-unit activity (MUA), yielding several advantages over LFP recordings, including sharper frequency tunings. ECoG first spike latency showed highest similarity to superficial PEA contacts and MUA traces maximally correlated with PEA recordings from the granular layer. These results confirm high quality of the ECoG grid recordings and the possibility to collect LFP and MUA simultaneously.

## Introduction

An important goal in neuroscience is to understand the activation patterns of neuronal networks at a high spatial and temporal resolution. This knowledge is important in a variety of medical applications, such as hearing restoration via cochlear implants. Both following auditory deprivation and restoration of hearing, spatio-temporal activation patterns of the auditory pathway are substantially altered^1^. The characterization of the spatio-temporal activation patterns in the auditory cortex has commonly been assessed via recordings with penetrating multi-electrode arrays (MEAs), which have the disadvantage of damaging the brain tissue^2^. This is especially disadvantageous for chronic recordings in behaving animals^2,3^. Different MEAs can be used that cover a wide range of temporal and spatial resolutions (~10 µm to 10 cm; for review see Lebedev & Nicolelis, 2017)^4^. One version of MEAs is the laminar single-shank microelectrode array used for comparison in this study, which allows conclusions on single cell activity^5^. In order to track ongoing changes in a chronic setting, for example after deprivation and following sensory restoration, spatially and temporally precise but non-invasive recording methods are favorable. Both local field potentials (LFPs) and action potential related activity are important to understand the complex neuronal responses to sensory stimulation^6^. LFPs are commonly assumed to reflect the input to the dendritic field, generated by synchronized synaptic activity, however, several electrical discharges may add to these slow potentials (<100 Hz) including Na^+^ and Ca^2+^ spikes, ionic fluxes through voltage- and ligand-gated channels, and intrinsic membrane oscillations^6,7^. The outputs of the respective neuronal tissue are action potentials, recorded extracellularly either as single spikes or as multi-unit activity (MUA), if they are recorded in some distance from the active cells. If the number of underlying cells cannot be resolved due to small amplitude and/ or high overlap, this spiking activity (>300 Hz) is sometimes referred to as “hash”^6^. LFP and spiking activity are not mutually exclusive measures: First, LFPs indicate events that are causal to action potentials^8^ and secondly, MUA activity is highly correlated with high-gamma (80–200 Hz) power of the brain oscillations^9^. It has been shown that spatial characteristics of cortical responses, such as tonotopy, are represented at a finer spatial resolution by spiking activity than by slow wave local field potentials (LFPs) recorded with the same electrodes. This is due to the fact that the effect of volume conduction is higher for LFPs and the small-amplitude, high frequency spiking activity diminishes faster with distance^8,10–12^ (but see Herreras, 2016)^13^. However, spiking activity is commonly only assessable using penetrating electrode arrays (PEAs). Subdural surface grids used in electrocorticography (ECoG) permit simultaneous recording of multiple sites over a large area of the cortex without the need for repeated (i.e. time consuming) penetrations of the brain tissue. But the combined effects of large distance to the neuronal tissue, large electrode contact size and high impedances (>1 MΩ at 1 kHz) usually limit surface recordings to local field potentials (LFP) and reduce spatial selectivity (usually >1 mm)^4,14^.

MUA recorded from the surface of the neocortex is mainly associated with activity of layer 1 interneurons as well as pyramidal cells and interneurons in deeper layers^15^. In the study of Khodagoly and colleagues^15^, isolation of single neuron action potentials was possible due to the neuron size, electrode diameter (10 µm) and the use of an organic interface material that conducts not only electric, but also ionic current. However, gold or platinum contacts are used in conventional ECoG recordings for both humans and animals^14,16,17^. Downscaled µECoG electrode grids for human use, with 1-2 mm electrode diameter and -spacing, enable LFP recording which are of a comparable spatial resolution to those of PEAs (i.e. several hundred µm)^14^. For animal studies, surface grids with lower impedances (~200 kOhm) and smaller electrode diameters (150 µm) have been developed^18,19^. Additionally, the introduction of thin-film technology enables intimate electrode-tissue coupling for precise and reliable mapping of spatial activation and thus is also well suited for chronic recordings^15,18,19^. These have already been shown to be suitable to detect MUA, in recordings from the basal root ganglia^20^. Based on positive findings in cats, but not humans, the authors conclude that the intimate electrode-tissue coupling is a key factor for surface MUA recordings and that low impedances and a flexible substrate material are critical for high recording quality.

In the present study a thin-film surface ECoG grid was developed in cooperation with Blackrock Microsystems, Europe, based on the current knowledge in the field. The grids were then evaluated for their potential to record spontaneous and evoked cortical activity. As there is currently no standard ECoG electrode established for animal research, the data was compared to a conventional single-shank PEA (‘Michigan probe’)^17^. We found that the new ECoG grids were suitable for recording both LFPs at high spatial resolution and MUA comparable to simultaneous recordings from PEAs.

## Materials and Methods

### Animals and surgical preparation

The experiments were performed in 14 (2 female) Dunkin-Hartley (albino) guinea pigs (372 ± 48 g). All procedures were in accordance with the German and European Union guidelines for animal welfare (ETS 123, Directive 2010/63/EU) and were approved by the German state authority (Lower Saxony state office for consumer protection and food safety, LAVES; approval No. 14/1514). Normal hearing was confirmed by auditory brainstem responses (ABRs; see acoustic stimulation for details).

Anesthesia was induced by an intra muscular injection of a combination of ketamine (50 mg/kg BW), xylazine (10 mg/kg) and atropine sulfate (0.1 mg/kg). For subsequent inhalation anesthesia, a custom-made endotracheal tube was inserted through a tracheotomy and connected to a ventilator (Rodent Ventilator 7025, Ugo Basile, Comerio, Italy). After surgical preparation, an adequate anesthesia level for cortical recordings was maintained by <1.5% isoflurane in O_2_/air and was surveyed by testing for paw-withdrawal and corneal reflexes. Vital functions were assessed by electro-cardiography (ECG) and capnometry (end-tidal CO_2_ vol%; Normocap CO_2_ & O_2_ Monitor, Datex, Helsinki, Finland). Body core temperature was kept at ~38.0 °C using a heating pad, controlled via feedback from a rectal probe (TC-1000 Temperature Controller, CWE Inc., Ardmore, USA).

For fixation in a stereotaxic frame (Stereotaxic Frame 1430, David Kopf Instruments, Tujunga, USA), the skull was exposed and a head-holder (custom, stainless steel fixation-rod) was secured to the bone anterior to suture-point Bregma using 3 bone screws (Ø 0.85 mm, Fine Science Tools GmbH, Heidelberg, Germany) covered with dental acrylic cement (Paladur, Heraeus Kulzer GmbH, Dormagen, Germany). For recordings from the auditory cortex, a unilateral craniotomy (~5 × 5 mm) was performed, centered at ~2.5 mm caudally from Bregma and 7.3 mm laterally from the midline. After removing the *dura mater* and positioning of the recording electrodes the brain was covered with medical grade silicone oil (M 5000, Carl Roth GmbH & Co. KG, Karlsruhe, Germany) to prevent dehydration through evaporation.

### Recording electrodes

The size of the ECoG grid (4 × 4 contacts, 4 mm^2^ surface area; 0.5 mm contact spacing) was chosen to cover the average area of the primary auditory cortex (A1) of the guinea pig. The substrate for the grid was a 20 µm thin parylene C film. The parylene C film was metallized with gold, which was photoresist-coated and developed, following direct writing (DWL 66 + , Heidelberg Instruments). After etching of gold and removal of photoresist residuals the grid was connected to a standard Omnetics connector to enable connection to the recording system. The final grids had 16 AU contacts (100 µm in diameter) with impedances of approximately 200 kΩ (see results). Defined openings in the parylene C substrate enabled simultaneous insertion of a PEA (A1x16-5mm-150-177-A16, NeuroNexus, Ann Arbor, USA). For the characterization of responses to broad-band stimuli, we recorded simultaneously from 14 cortical positions (12 animals) with 244 recording sites for the ECoG grid and the PEA, respectively. To assess spatial selectivity in terms of tonotopy, we recorded from 21 surface positions overall (N = 14 animals, N = 336 ECoG recording sites) in combination with 18 positions from a PEA with 16 channels and impedances of 1.0 ± 0.1 MΩ at 1 kHz (N = 288 recording sites). A silver ball electrode covered in salt-free electrode gel (Spectra 360, Parker Laboratories INC., New Jersey, USA) was placed through a hole ~1 mm rostral from Bregma onto the *dura mater* as a recording reference for both the ECoG grid and the PEA It was sealed against leakage of cerebro spinal fluid with surgical tissue adhesive (Histoacryl, B. Braun Melsungen AG, Melsungen, Germany) and covered with dental acrylic cement.

### Acoustic stimulation

Prior to surgery, ABR click thresholds (i.e. lowest sound intensity inducing a visual discernable response) were assessed via two trans-dermal silver wire recording electrodes: one at the vertex and one as retro-auricular reference electrode. The 50 µs condensation clicks were presented via an audiometric headphone speaker (DT48, Beyerdynamic, Heilbronn, Germany) in fifteen 5-dB steps (sound pressure level indicated as peak equivalent: 26–91 dB_SPL pe_), with 50 repetitions each. Normal hearing was assumed for ABR thresholds of ≤35 dB_SPL pe_.

All other acoustic stimuli were presented in randomized order with 30 repetitions each (recording interval: 827 ms). Stimuli were delivered via the audiometric headphone speaker to the ear contralateral to the cortical recording side. The stimulation was performed in closed field condition, using a custom build polyvinyl chloride cone, fixed to the speaker and set onto the external meatus. The responsiveness to broad-band stimuli was accessed using 100 ms white noise bursts (10 ms cosine ramps), presented in seventeen 5-dB steps (sound pressure level indicated as root mean square value: 0–80 dB_SPL rms_). To describe the tonotopic organization of A1, pure tones (100 ms, 10 ms cosine-ramps) at frequencies between 1 kHz and 32 kHz with usually 0.5 octave increments were presented in nine 10-dB steps (0–80 dB_SPL_). The sound levels were calibrated prior to the experiments at the tip of the stimulation cone through the stimulation software (AudiologyLab, Otoconsult, Frankfurt a.M., Gemany) using a condenser microphone (1/4″ microphone [4939] in combination with a preamplifier [2670] and a Nexus conditioning amplifier [2690], Brüel & Kjaer, Nærum, Denmark).

### Data recording and analysis

Recordings of both the ECoG grid and the PEA were performed using a custom build recording setup and software (AudiologyLab, Otoconsult, Frankfurt, Germany). The signals were acquired and amplified through a multichannel recording system (Lynx-8 amplifier system, amplification 8000 or 5000 times, butterworth filter: 1 Hz–9 kHz, rolloff: 12 dB per octave, Neuralynx, Bozeman, USA) and stored through AudiologyLab at a sampling rate of 25 kHz using a 32-channel MIO card (NI-6259 National Instruments, Austin, USA). The three deepest PEA contacts were excluded from the analysis, as they were commonly inserted beyond the grey matter of the AC due to the length of the PEA. These contacts usually did either show no responses or did not record activity above the threshold criteria described below.

The peak to peak amplitude of the LFP (resampled at 2 kHz via a Matlab routine) was analyzed from 0–200 ms after stimulus onset. MUA spike rates (spikes/stimulus/ms) were measured from 10–40 ms after stimulus onset. MUA was calculated as described previously^21^. In short, the signal was filtered (zero-lack, 2nd order elliptic filter: 300–3000 Hz) and spikes with at least 0.08 ms duration above the detection threshold (i.e. 3* standard deviation, SD, based on median activity of the signal)^22^ were counted as MUA. The level of background activity was measured during a 50 ms pre-stimulus time window and was subtracted from the respective measure (LFP amplitude or MUA rate) during the post-stimulus time. For comparison of onset-response timing between ECoG grid and PEA, the first-spike latency (FSL) was calculated from stimulus onset, as the time when the given spike train differed significantly (p < 0.001) from a Poisson distribution^23^.

As measure of overall responsiveness, we described input-output functions for broad-band noise stimuli. We excluded data sets as ‘non-responsive’ for which the highest sound intensity did not evoke at least twice the average background activity, calculated as mean value over all intensities for each contact. We fitted the functions with a sigmoidal fit (e.g. Konerding *et al*., 2017)^24^ and derived the response threshold (i.e. sound intensity [dB] inducing 10% of maximal response amplitude) and dynamic range (90% of max–10% of max). In individual cases, the highest sound intensity (80 dB_SPL rms_) induced lower response amplitude than the second highest (75 dB_SPL rms_), potentially due to the middle ear muscle reflexes^25^. In these cases, the fitting was performed without including the responses to 80 dB_SPL rms_. To gain a comparable measure of response strength for MUA and LFP, we calculated the response-background ratio (RBR) at 20 dB above response threshold as dB above the average background activity. As the MUA is mainly a binary measure, we assessed the RBR in terms of changes in spike rate above background MUA rate^26^.

The tonotopic organization of the auditory cortex was determined based on the characteristic frequency (CF, frequency that elicits responses at lowest sound intensities) for each recording site. To calculate the CF, we interpolated a tuning curve at 10% of the maximal LFP amplitude or MUA rate of a given contact^27^, if the maximum exceeded 3 times the standard deviation of the background activity. The tuning width was calculated in terms of Q20-values: CF divided by the bandwidth of the tuning curve 20 dB above the sound intensity at CF (from here: CF threshold). For visual inspection of the tonotopy, we defined CFs with a resolution of 1 octave and plotted all contacts, spatially aligned based on arterial and suture landmarks. Based on the border of A1 to the dorsal cortex (DC), as inferred from a frequency reversal, we defined the tonotopic axis orthogonal to the border. To compensate for inter-individual differences in cortex structure, we normalized the data according to the coordinates of the 8 kHz CF (taking the mean if there were several sites with 8 kHz for one individual). Subsequently, a linear regression was calculated along this tonotopic axis to assess the spatial tonotopy resolution in mm/octave, for each individual and for the whole data set.

**Figure 1:**
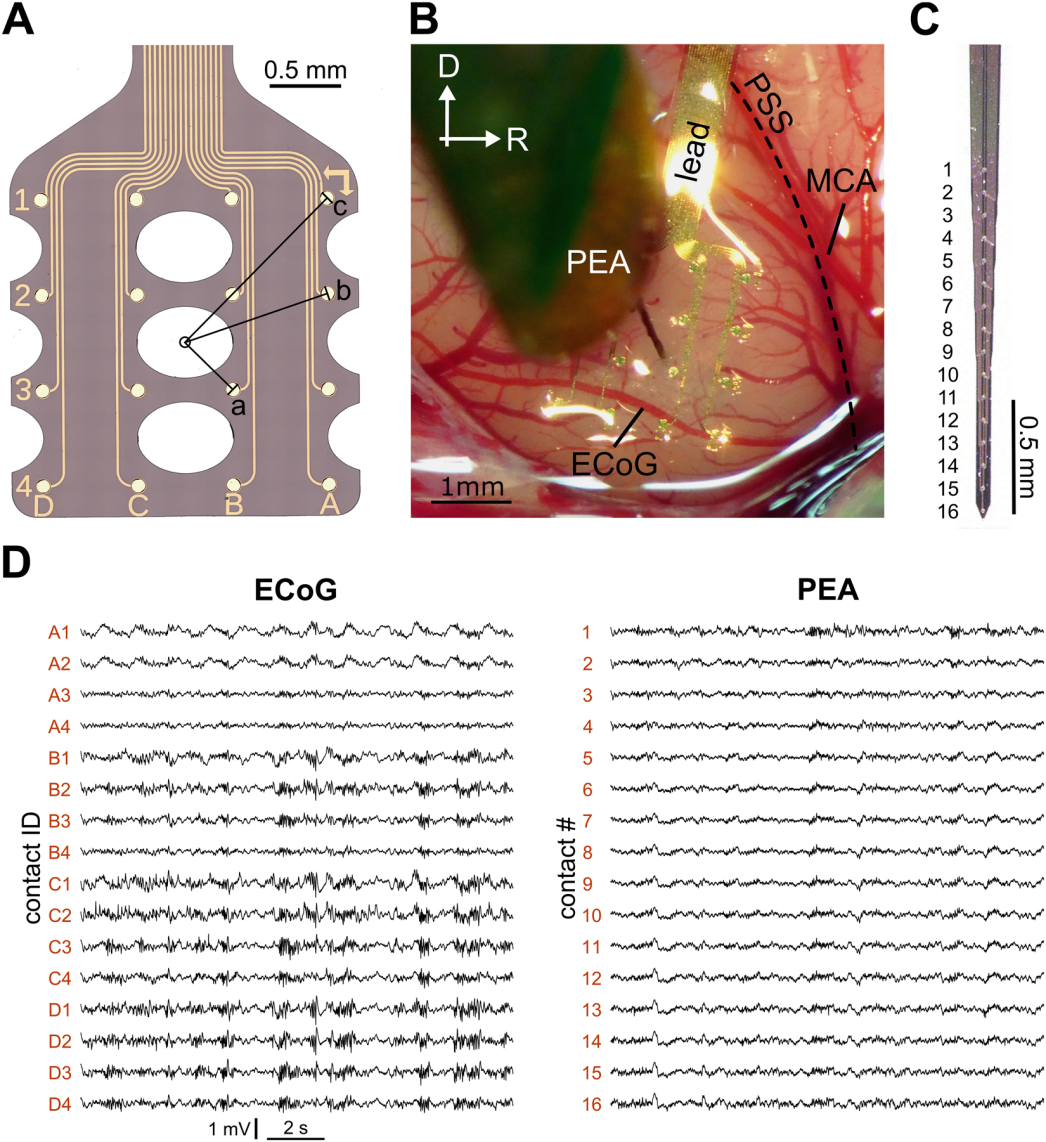
Recordings from the newly developed ECoG grid were possible for all 16 surface contacts and were similar to simultaneous recordings from a conventional penetrating multi-electrode array. (**A**) Sketch of the subdural ECoG grid. Markers on the grid facilitate the allocation to the respective surface locations. The distances to the central insertion point are indicated (a = 0.5 mm, b = 1.0 mm, c = 1.3 mm) (**B**) The penetrating multi-electrode array (PEA) is inserted through an opening in the ECoG grid substrate. Marked are the pseudo sylvian sulcus (PSS) and the middle cerebral artery (MCA), which served as landmarks for spatial alignment of different recording positions. (**C**) Example image of a 16 channel single shank PEA. (**D**) Examples of traces (raw signal) for an ECoG grid and a penetrating multi-electrode array from the same cortical recording during spontaneous activity.

The subdural ECoG grid was compared to the PEA with regard to both horizontal and laminar distance. For the CF distribution along the surface, the PEA contact with lowest CF threshold is compared to the ECoG grid contacts pooled according to distance from the insertion point: close = 0.5 mm (i.e. 4 inner contacts), medium = 1 mm, far = 1.3 mm (Fig. 1A). For every distance, the CF difference between ECoG grid and PEA contacts is calculated in octaves. If several PEA contacts showed the same low CF threshold, the median CF was chosen (two data sets excluded, as no CF was discernable at the PEA). To estimate the laminar contribution to the ECoG recordings, we assessed the similarity in FSL and the correlation strength. The FSL was calculated for the best frequency (BF). The BF was defined as the frequency that elicited highest summed spike rates over all sound levels. It was used as non-interpolated estimate of the CF (based on MUA), since both measures were highly correlated (spearman correlation: p < 0.001, r = 0.915 N = 317; Supplement Fig. 1). We used the mean FSL of the four ECoG contacts surrounding the insertion point and pooled the PEA contacts based on the estimated location in different cortical layers: supragranular layers: 0–600 µm (contact #1–5), granular layer: 800–1100 µm (contact #6–9), infragranular layers: 1350–1800 µm (contact #10–13). The correlation between the ECoG grid recordings and the PEA was assessed in response to noise bursts to exclude the influence of frequency selectivity. The correlation strength was assessed as two-dimensional (time and repetition) Pearson correlation (r^2^) between the 4 ECoG contacts closest to the insertion point and all 16 PEA contacts, using single sweeps (autocorrelation: r^2^ = 1). Recordings were excluded whenever clipping was observed in more than 2 individual sweeps. To assess the correlation of LFPs, we analyzed the raw signals in a time window of 0–200 ms from stimulus onset. To correlate spiking activity at the surface and in the depth we transferred the MUA event train into a continuous signal by calculating the moving average (1 ms integration width). As an estimate of overall correlation between the ECoG grid and the PEA, we calculated a sweep-wise sum over all 16 PEA contacts. The surface contact with the highest correlation to this summed signal was used to analyze the effect of insertion depth, correlating each of the 16 channels, separately, with the respective ECoG contact.

### Statistical analysis

Considering the focus of this study and due to the difference in electrode contact characteristics (e.g. size and impedance), we assessed the recordings of the ECoG grid and PEA contacts as independent measures and performed unpaired analyzes for the comparison of LFP characteristics at both electrodes. We performed paired comparisons for LFP and MUA recorded at the same electrode contact. The Kolmogorov-Smirnov test was used to confirm normal distribution of the data and, if required, non-parametric analyzes were applied and the median ± interquartile range (IQR) instead of the mean ± standard deviation (SD) was calculated. The level of significance was set to 5%.

## Results

We first described the properties of the new ECoG grid and its potential for recording spontaneous activity from the auditory cortex. Subsequently, we showed the similarity of LFP recordings from the ECoG grid to simultaneously recorded LFP of PEAs. We assessed both the evoked responses to broad-band stimuli in terms of input-output functions and showed stimulus specificity in terms of tonotopy, using tonal stimulation. To explore the origin of the ECoG recordings, we furthermore correlated subdural recordings with recordings from different cortical depths. Finally, we described the potential of the ECoG grid to record MUA from the surface and related the derived measures to those of the LFP recordings.

### ECoG grid design and spontaneous activity recording

The grid substrate parylene C achieved high flexibility and enabled an intimate contact at the electrode-tissue interface. The average impedance was 212 ± 51 kΩ (measured at 1 kHz; n = 5 grids), ranging from 128 to 395 kΩ (one extreme value with 962 kΩ). Individual ECoG grids were used up to six times, without observable changes in recording quality. Simultaneous recordings from a PEA inserted through one of the openings in the grid material were possible with high signal quality (Fig. 1).

### Evoked LFP responses to broad-band stimuli

**Figure 2:**
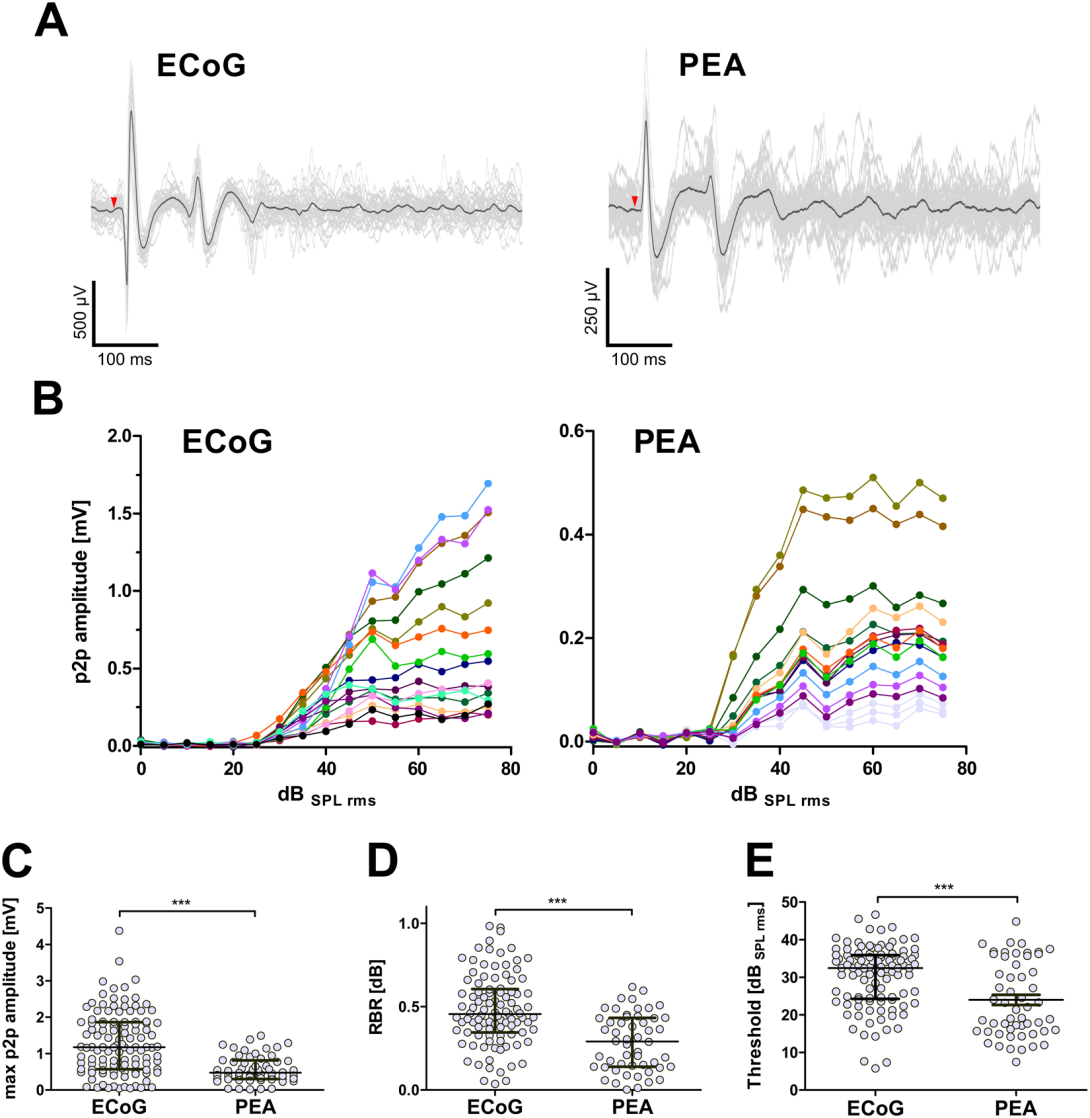
The new ECoG grid enabled recordings of evoked LFP responses similar to conventional penetrating multi-electrode arrays. (**A**) Examples of evoked responses to a 100 ms broad-band stimulus for an ECoG grid and a penetrating multi-electrode array (PEA) from the same cortical recording. Given are 30 single sweeps (thin grey lines) and an averaged signal (thick black line). Stimulus onset is indicated (red arrow head). (**B**) Input-output functions of LFP peak-to-peak (p2p) amplitudes in response to a broad-band stimulus. The colors represent different recording positions; the three deepest PEA contacts (excluded from further analysis) are indicated in light grey. The amplitudes at the penetrating electrode (PEA) are significantly smaller than those at the ECoG grid (C and D). (**E**) The response thresholds at ECoG grid contacts was slightly higher (i.e. worse) than those recorded at the penetrating electrode (PEA) contacts. (**C**) The maximal peak to peak (p2p) amplitude at ECoG grid contacts was significantly higher than those recorded at the penetrating electrode (PEA) contacts. (**D**) The response-background ratio (RBR) at 20 dB above response threshold was significantly higher at ECoG than at PEA contacts. (**C**–**E**) Given are individual data (dots) and medians with IQR (lines). Mann-Whitney U test: ***p < 0.0001.

To assess the potential of the new ECoG grid to record brain activity during sensory stimulation (Fig. 2A), we analyzed the evoked LFP responses to broad-band stimuli recorded with the ECoG grid and compared these to simultaneous recordings from PEAs at 14 cortical positions. Of 224 recording sites, 126 on the ECoG grid showed a clear onset response (56.3%; criterion: >2* background activity) while this was the case for 58 (31.9%) recording sites on the PEA (N = 182; 3 deepest contacts excluded). The input-output functions (Fig. 2B) showed some variability across recording sites (Fig. 2B), and the sigmoidal fit was not possible for 15 ECoG and 4 PEA responsive sites. In the remaining data sets, the goodness of fit was high (ECoG: r^2^ = 0.956, SD: 0.046, n = 111; PEA: r^2^ = 0.955, SD: 0.025, n = 54). The calculated maximal peak to peak amplitude was significantly higher for the ECoG (median: 1.177 mV) as compared to the PEA (0.478 mV; Mann-Whitney U test: p < 0.0001, U = 1475; Fig. 2C). The response-background ratio at 20 dB above response threshold was higher for the ECoG grid (median: 0.455, n = 101, negative values excluded) than for the PEA (median: 0.292, n = 52, negative values excluded; Mann-Whitney U test: p < 0.0001, U = 1308; Fig. 2D). The calculated response threshold was higher at ECoG contacts (median: 32.46 dB_SPL rms_) than at PEA contacts (21.75 dB_SPL rms_; Mann-Whitney U-test: p < 0.0001, U = 1817; Fig. 2E). The dynamic range was however similar for ECoG grid and PEA contacts (median_ECoG_: 29.79 dB; median_PEA_: 36.10 dB; Mann-Whitney U test: p = 0.369, U = 2738).

### Tonotopy based on LFP recordings

**Figure 3:**
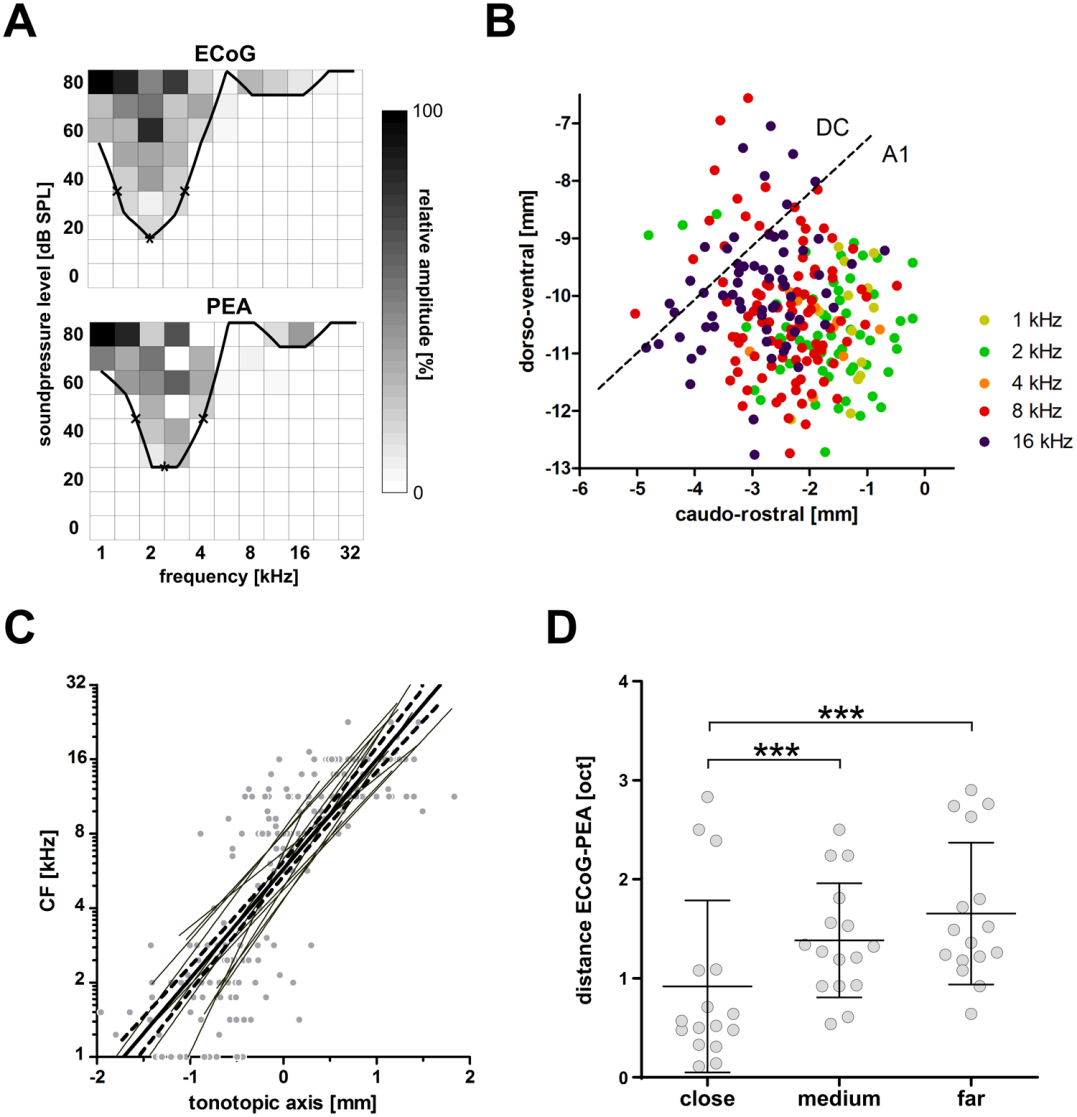
The ECoG grid enabled fine-scale tonotopic mapping of the GP auditory cortex based on LFP recordings. (**A**) Example of frequency response curves derived by ECoG grid recordings and recordings from penetrating electrodes. Indicated are the CF and the lower and upper value 20 dB above CF, from which Q20-values were calculated. (**B**) Tonotopic map of the auditory cortex. For each ECoG grid contact (N_LFP_ = 241) the CF in octave increments was plotted over the respective cortical coordinate. A frequency reversal (dashed line) marks the transition from the primary auditory (A1) to the dorsocaudal cortex (DC). (**C**) Correlation of CF with the tonotopic axis of A1 for LFP recordings from all ECoG grid recording sites. Given are individual data (dots) and regression lines per individual (gray, thin lines), as well as the overall regression (thick solid line) with the 95% confidence interval (dashed lines). (**D**) The CF distance in octaves between ECoG grid recording sites and penetrating multi-electrode array (PEA) increased with increasing surface distance. Given are individual data (dots) and mean with SD (lines). Repeated measure 1-way ANOVA p < 0.002 with Bonferroni post-test: ***p < 0.001.

To determine the spatial selectivity of recordings from the ECoG grid, we assessed the frequency selectivity of each recording site and the change in characteristic frequency (CF) with cortical position (i.e. tonotopy). For each recording site at the ECoG grid (N = 336) and at the PEA (N = 234), we determined the CF and the tuning width in terms of Q20-values from the frequency response curves based on LFP recordings (Fig. 3A). Based on the defined CF threshold (criterion see methods), 12% of the ECoG recording sites were defined unresponsive and were excluded from the analysis. In another 10% no single CF could be determined, due to the double-peaked appearance of the frequency tuning curve (FTC). Additionally, there was 1 case in which the frequency response area showed no clear FTC and in 15 cases the CF was at or possibly below 1 kHz (lowest stimulated frequency) and no Q20-value could be assessed. Of the PEA recordings, 41% were defined unresponsive (i.e. fell below CF threshold criterion), 13% of the FTCs were double peaked, 3% had a CF at 1 kHz and 1 case (not the same animal as for the ECoG grid) had a complex response characteristic.

The average Q20 tuning width was similar for the surface and penetrating electrodes (median_ECoG_ = 0.995, median_PEA_ = 0.916; Mann-Whitney U test: p = 0.361, U = 11411, n_ECoG_ = 246, n_PEA_ = 99). This difference was not based on a differences in CF, as ECoG and PEA contacts had similar CF values (median_ECOG_ = 8.1, median_PEA_ = 8.0; p = 0.797, U = 26381, n_ECoG_ = 261, n_PEA_ = 205). The spatial distribution of CF of the combined ECoG recording sites showed a frequency reversal, which marks the transition from the primary auditory cortex (A1) to the dorsocaudal cortex (DC, Fig. 3B).

Based on a virtual line of the medio-lateral frequency reversal we defined the orthogonal, rostro-caudal tonotopic axis. All CFs within A1 were correlated with their cortical coordinates relative to the tonotopic axis and for each individual we derived the slope of the resulting linear regression (Fig. 3C). One animal was excluded due to very poor fitting (r^2^ = 0.057), the remaining 13 datasets had a high coefficient of determination (r^2^ = 0.709, SD: 0.139). The slope for all data was 0.678 mm/octave (r^2^ = 0.685). The individual slopes had a low spread around the mean (0.646 mm/octave) with a standard deviation of 0.153 mm/octave and a range from 0.373 to 1.000 mm/octave.

Correspondingly, the CF difference between the ECoG and PEA contacts was around 1 octave for contacts close (0.5 mm) to the insertion point (mean ± SD: 0.918 ± 0.869 octave) and significantly increased with horizontal distance (repeated measure 1-way ANOVA: p < 0.0001, n = 16; Fig. 3D).

#### Correlation with laminar distance

**Figure 4:**
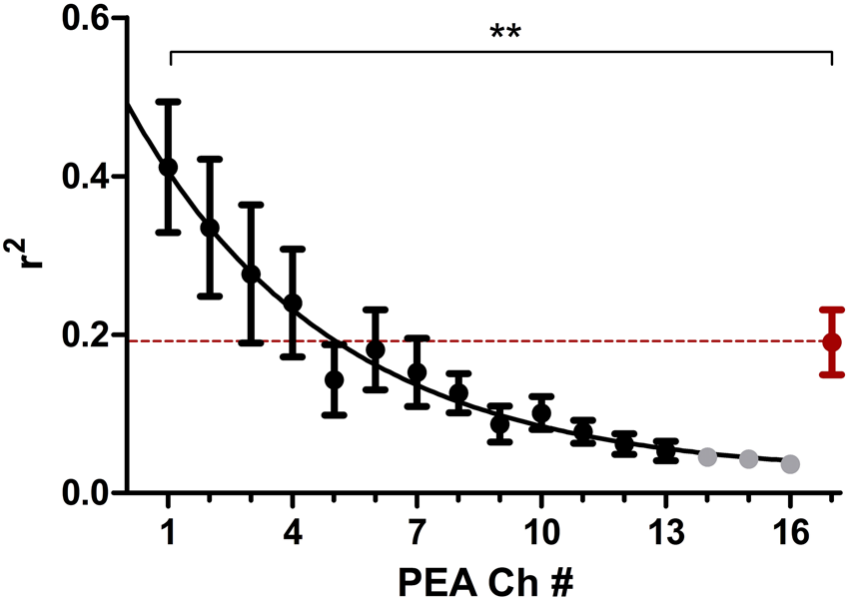
Correlation strength (r^2^) between grid and penetrating electrode recordings for LFP. Given are mean (dot) and SEM (whisker) and the non-linear regression line (One-phase exponential decay: r^2^ = 0.370). Based on the raw signal, we revealed that recordings of the grid and the penetrating (PEA) contacts significantly declined with cortical depth (contacts #14–16, indicated in light grey, were excluded from further analyses). Beyond 5 contacts (~ 750 µm), r^2^ fell below the summed sweep-by-sweep correlation strength over all 16 penetrating contacts (red). ANOVA with Bonferroni corrected posttest: **p < 0.01.

In order to determine the origin of the LFP signals recorded at the ECoG grid, we correlated the signal with those recorded via the PEA. The correlation strength (r^2^) significantly declined with cortical depth from channel #1 to #16, with a high correlation for the most superficial channel #1 (r^2^ = 0.411) and weakest correlation for channel #16 (r^2^ = 0.036; 1-way repeated measure ANOVA: p < 0.0001, F = 8.426, df = 15; Fig. 4). The comparison with the correlation strength between ECoG grid and summed signal of the PEA (r^2^ = 0.190) revealed that only the signal recorded at the first contact showed a significant higher correlation with the ECoG grid recordings. The summed signal of the PEA explained less variance of the ECoG recordings than each of the 4 superficial PEA contacts, individually.

#### Comparison of MUA activity at the ECoG grid to the PEA

**Figure 5:**
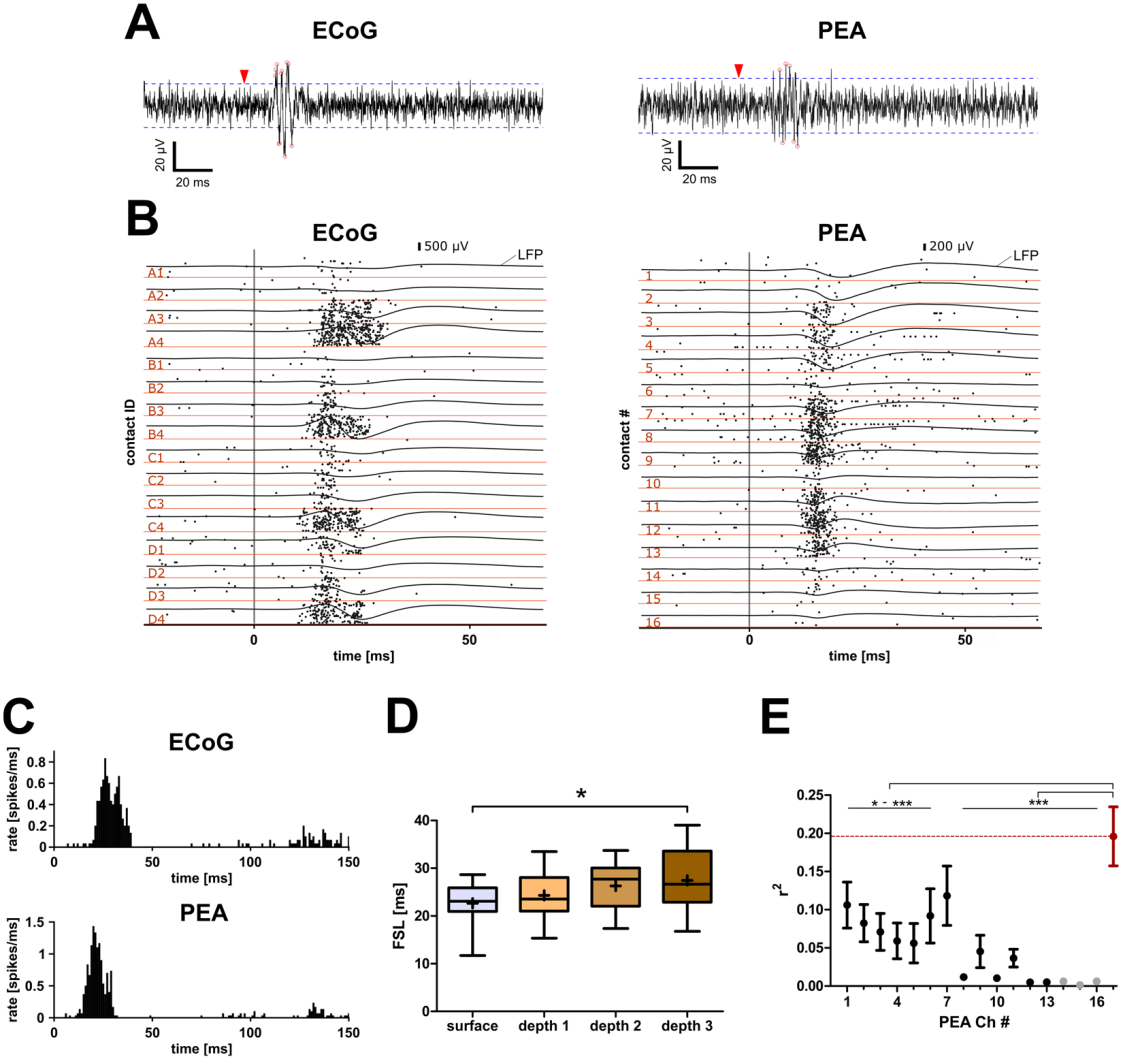
The newly developed ECoG grid enabled recordings of evoked MUA similar to conventional penetrating multi-electrode arrays. (**A**) Examples of filtered (300–3000 Hz) traces with detected spikes (red circles) above detection threshold (dashed line) for both ECoG and penetrating multi-electrode array (PEA). Stimulus onset is indicated (red arrow head). (**B**) Representative examples of averaged LFP and raster-plots of MUA in response to a broad-band noise stimulus of 80 dB_SPL rms_. The raster-plots indicate the time points of every detected spike in each of the 30 repetitions. Stimulus onset is indicated (vertical line). (**C**) The peri-stimulus time histograms (psth) show representative responses to a 100 ms broad-band stimulus, for the ECoG grid and the penetrating electrode (PEA), respectively. Usually a strong onset response and a weak offset response were discernable. (**D**) Similarity between first spike latency (FSL) for ECoG grid and penetrating multi-electrode array contacts decreases with cortical depth (depth3 = infragranular layer). Given are median FSLs at the best frequency threshold level (i.e. 10% of max MUA, comparable to the CF threshold) for ECoG grid recording sites close to the insertion point and for different depths at the PEA (depth 1: 0–600 µm, depth 2: 750–1200 µm, depth 3: 1350–1800 µm). Box-plots with min and max values (whisker) and mean (cross). The FSL at the surface was significantly shorter than the one recorded in deep layers of the AC; dependent t-tests with Bonferroni correction *p < 0.05. (**E**) Correlation strength (r^2^) between grid and penetrating electrode recordings for MUA is highest for granular layers (contact #7). Given are mean (dot) and SEM (whisker). After an initial decline in correlation strength, the maximal correlation was reached at the 7^th^ contact and sharply declined with increasing cortical depth (contacts #14–16, indicated in light grey, were excluded from further analyses). ANOVA with Bonferroni corrected posttest: *p < 0.05, **p < 0.01, ***p < 0.001.

We analyzed the high frequency component (300–3000 Hz) of the recorded signal that appeared as hash in the single traces (Fig. 5). From 224 and 182 recording sites at the ECoG grid and the PEA, respectively, 193 (86.2%) and 83 (45.6%) were defined responsive to broad-band stimuli based on a MUA rate (Fig. 5) of at least twice the background activity. The MUA had a clear onset response with a sigmoidal input-output function (Fig. 5B). Usually, also a weak offset response was discernable (Fig. 5C, data not analyzed).

To determine the potential origin of MUA recorded at the ECoG grid, we compared the FSL between ECoG and PEA contacts and also derived the correlation strength between the respective signals. The mean FSL (22.65 ms) of the four surface contacts surrounding the insertion point was similar to those recorded by superficial contacts of the PEA (depth 1/ supragranular layer: 24.33 ms, depth 2/ granular layer: 26.27 ms) and differed significantly from the one in deeper, infragranular layers of the cortex (depth 3: 27.45 ms; dependent t-tests with Bonferroni correction: surface vs depth1 p = 0.792; surface vs depth2 p = 0.342; surface vs depth3 p = 0.012, N = 15, n = 3 comparisons; Fig. 5C).

The correlation strength of the MUA between the ECoG and the PEA contacts showed significant changes with cortical depth (ANOVA: p < 0.001, F = 4.422, df = 15; Fig. 5D). The summed signal over all PEA contacts yielded a significantly higher correlation strength with the ECoG grid (r^2^ = 0.191) than most of the individual contacts. Only the correlation strength between the ECoG grid and the 7^th^ contact (~900 µm deep, r^2^ = 0.142) was not significantly different from the one with the summed signal and also had significant higher values than most of the electrode contacts inserted deeper into the cortex. Thus, the summed signal of the PEA did not explain significantly more variance in the surface recordings than the penetrating contact #7 on its own.

#### Comparison between MUA and LFP measures at the same ECoG grid and PEA contacts

**Figure 6:**
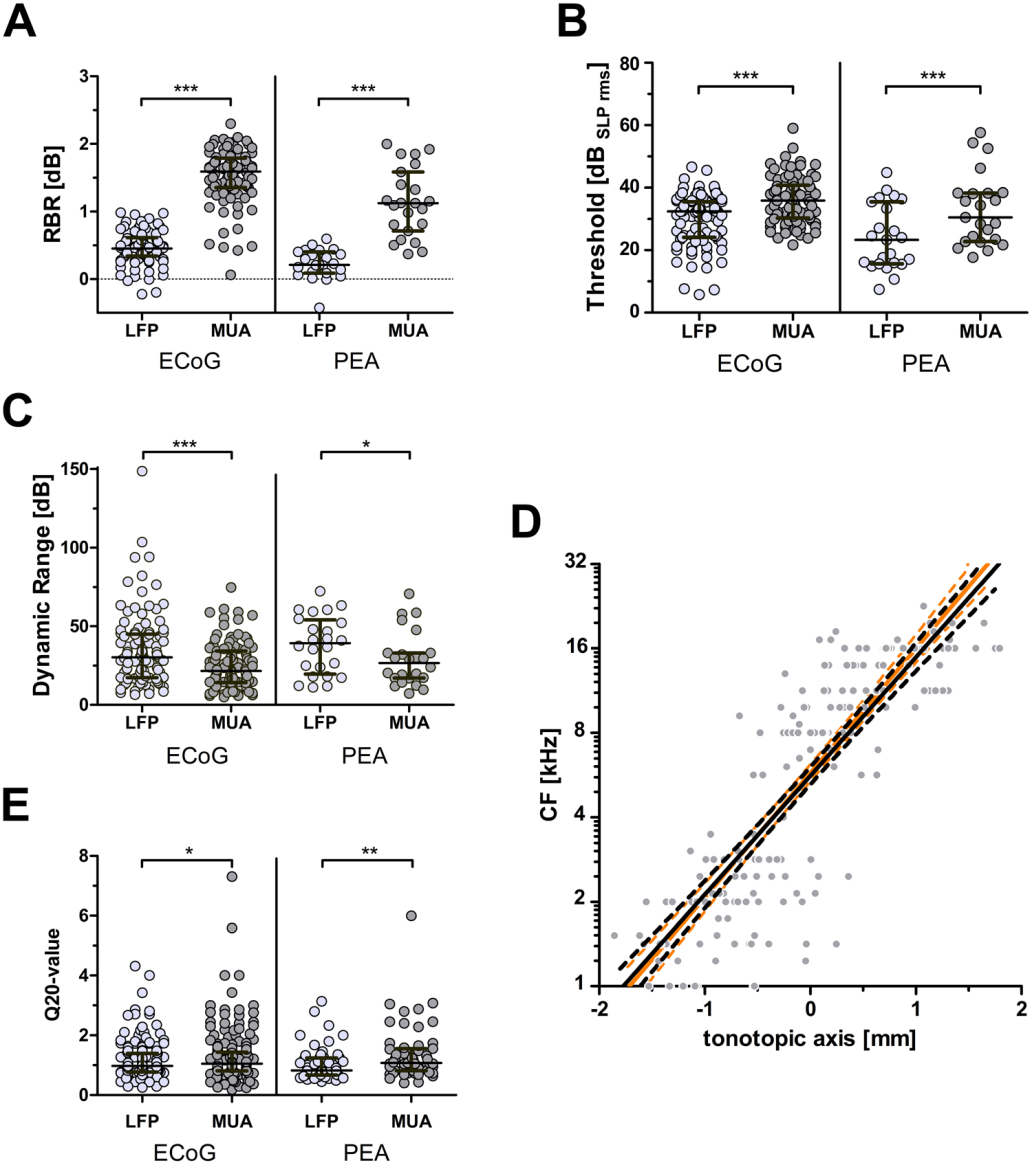
The characterization of the surface responses differed significantly between MUA and LFP measures at the same contacts. (**A**) The response-background ratio (RBR) 20 dB above response threshold was significantly higher (i.e. better) for MUA (based on rate) as compared to LFP (based on amplitude). Given are individual data (dots) and medians with IQR (lines). Wilcoxon test: ***p < 0.0001. (**B**) The response threshold was significantly higher (i.e. worse) for MUA as compared to LFP measures. Given are individual data (dots) and medians with IQR (lines). Wilcoxon test: ***p < 0.0001. (**C**) The dynamic range was significantly lower for MUA as compared to LFP measures. Given are individual data (dots) and medians with IQR (lines). Wilcoxon test: *p < 0.05, ***p < 0.0001. (**D**) The MUA Q20-values were significantly larger (i.e. sharper tuning) compared to LFP Q20-values. Given are individual data (dots) and median with IQR (lines). Wilcoxon test: *p < 0.05, ***p < 0.0001. (**E**) The tonotopy derived by MUA measures (black) at the ECoG grid was similar to the one derived by LFP measures (light orange). Given are individual data (dots) and the linear regression with the 95% confidence interval (solid and dashed lines).

To assess whether the MUA assessment gave information in addition to the LFP measures, we first compared the activity to broad-band stimuli with regard to the response threshold, dynamic range and the response-background ratio (based on LFP amplitude or MUA rate) at 20 dB above response threshold. The sigmoidal fit of the MUA input-output functions was not possible for all contacts. The respective sample sizes for paired data (i.e. LFP and MUA at the same contact) are given below. The results were not specific to the ECoG grid, but were also found for the PEA. The response-background ratio was significantly higher for MUA (ECoG: 1.589, PEA: 1.122) compared to LFP measures (ECoG: 0.451, PEA: 0.209; Wilcoxon test: ECoG: p < 0.0001, W = −4465, n = 94; PEA: p < 0.0001, W = −276, n = 23; Fig. 6A). The response threshold was significantly higher for MUA (ECoG: 35.87 dB_SPL rms_, PEA: 30.49 dB_SPL rms_) compared to LFP measures (ECoG: 32.40 dB_SPL rms_, PEA: 23.29 dB_SPL rms_; Wilcoxon test: ECoG: p < 0.0001, W = −37490, n = 94; PEA: p = 0.0002, W = −242, n = 23; Fig. 6B). The dynamic range was significantly lower for MUA (ECoG: 21.58 dB, PEA: 26.62 dB) compared to the LFP (ECoG: 30.27 dB, PEA: 39.18 dB; Wilcoxon test: ECoG: p < 0.0001, W = 3049, n = 102; PEA: p = 0.029, W = 154, n = 24; Fig. 6C).

To assess whether the spatial selectivity of the ECoG grid MUA recordings was higher than the one derived from LFP recordings, we compared the frequency selectivity measures CF slope along the tonotopic axis and the Q20-values. The slope for all MUA data was 0.712 mm/octave and individual slopes (min: 0.419, max: 1.890 mm/octave) did not differ significantly from those derived by LFP measures (Wilcoxon signed rank test: p = 0.588, W = −17, n = 13 animals; Fig. 6D). As for the LFP recordings, the CF difference between the ECoG and PEA was below 1 octave for contacts close (0.5 mm) to the insertion point (mean ± SD: 0.937 ± 0.710 octave) and significantly increased with increasing distance (repeated measure 1-way ANOVA: p = 0.010, n = 16). The MUA Q20 tuning width was similar for ECoG and penetrating electrodes (median_ECoG_ = 1.043, median_PEA_ = 1.073; p = 0.343, U = 10255, n_ECoG_ = 239, n_PEA_ = 92). However, when comparing the LFP tuning widths to those of the MUA, the MUA Q20-values (ECoG: 1.053, PEA: 1.075) were significantly larger than the LFP Q20-values (ECoG: 0.979, PEA: 0.823), indicating a slightly higher spatial resolution derived by MUA. This was the case both for the PEA (Wilcoxon signed rank test: p = 0.009, W = −663, n = 55) and for the ECoG (p = 0.040, W = −2918, n = 206; Fig. 6E). This difference in tuning width was not based on a difference in CF (with higher CF having higher Q-values), as the corresponding CF-values were similar or even lower for MUA than for LFP measures (ECoG: median_LFP_: 8.1, median_MUA_: 8.1, p = 0.816, W = −229, n = 220; PEA: median_LFP_: 6.8, median_MUA_: 2.4, p < 0.001, W = 1366, n = 71).

## Discussion

Subdural ECoG grid recordings allow scanning large brain areas by using multiple recording sites, without damaging the underlying brain tissue. The newly developed ECoG grid (Blackrock Microsystems Europe) was found to yield similar recording quality as a conventional PEA (i.e. Neuronexus ‘Michigan probe’). The impedances of the ECoG grids were low (mean 212 kΩ) and showed little variability between contacts (SD: 51 kΩ; one extreme at 962 kΩ). The thin-film material allowed intimate contact to the brain surface and recordings of spontaneous brain activity could be obtained from all ECoG grid contacts. Recording quality was not compromised by covering the grid and brain by silicone oil. Evoked responses could be reliably assessed in a vast majority of the recording sites and the incidence of non-responsive sites was much lower than for the PEA for both broad-band stimuli (26 vs. 54%) and pure tones (13 vs. 47%). Although we did not systematically assess the stability of the ECoG grid over several recording sessions, we observed persisting high recording quality for up to six experiments, using the same ECoG grid. We found that these new ECoG grids were suitable for recording both LFPs at high spatial resolution and MUA comparable to conventional PEAs.

By recording simultaneously from the surface ECoG grid and the PEA, we could directly compare evoked LFP responses between the two electrode types. The ECoG grid LFPs were on average larger, had a higher response-background ratio and showed slightly elevated response thresholds as compared to the PEA recordings. The dynamic range was similar between the electrode types.

One potential explanation for significant differences between surface and penetrating electrode recordings would be differences in cortical depth. However, we did not find any indications in our data (analyzes not shown) and to the best of our knowledge, no such difference have been reported in the literature^28^. The difference in response threshold was unexpected and the distribution of values highly overlapped between the two electrode types. A different involvement of input sources, including long latency activity, may have led to a higher variance in individual surface recordings and the observed small increase of ECoG grid response thresholds in the averaged signal, as compared to the depth recordings. Larger amplitudes at the ECoG grid contacts as compared to the PEA may arise due to lower impedances (200 Ωk vs 1 ΩM). However, the influence of impedance values and electrode contact geometry is discussed to be rather negligible for LFP recordings^29^. Additionally, larger amplitudes and higher response-background ratios may arise from recordings from a higher number of synchronously active neurons^7^. This would indicate that integration occurs over more neurons at the ECoG grid electrode as compared to the penetrating electrode contacts. However, the high spatial acuity and similarity in tuning width (see below) showed that the ECoG grid contacts did not integrate over a significantly larger amount of cells, at least in the horizontal plain. Furthermore, the size of LFP amplitudes is discussed to be influenced by the cytoarchitectonic structure rather than by functional factors^13^. The fact that the surface electrode is recording from the outside of the activated tissue, in a different orientation to the LFP source(s), as compared to the PEA, may therefore explain the significant differences in LFP magnitude.

The ECoG grid enabled fast and reliable assessment of the tonotopic map, revealing the typical tonotopic organization of the auditory cortex with a frequency reversal defining the transition between A1 and DC. Most of the derived frequency tuning curves (FTC) were V-shaped and allowed the designation of a CF and calculation of Q20-values. Only in about 10% of both the surface and the depth recordings double-peaked FTC^30^ were found. This corresponds well to observations of uniform V-shapes under anesthesia with increasing numbers of multi-peaked FTC in awake animals, e.g. 20% of neurons in marmosets (for review see Schreiner *et al*., 2011)^31^. The individual frequency slopes along the tonotopic axis were highly consistent and the average slope was 0.68 mm/octave (0.71 mm/octave for MUA). This value is similar to the one reported by Hellweg and colleagues of 0.63 mm/octave^32^ and corresponds well to frequency distributions in A1 reported in the literature^15,30,33^. These were derived by multiple insertions of penetrating electrodes^30,32,33^ or by optical imaging methods^15^. Based on the guinea pigs broad hearing range of 0.05–50 kHz (~9.5 octaves)^34^ and the size of A1 of 4 mm along the tonotopic axis (Fig. 3C and^30^), a slope of 0.42 mm/octave would be required to evenly represent the whole frequency range. This theoretical slope is considerably steeper than the values derived by mapping the auditory cortex. The discrepancy may be based on under-representation of parts of the hearing range, or omissions of specific frequencies. The two effects have been described in several mammalian species and are both discussed to reflect specific environmental adaptations^31^. In our data, the distribution of frequencies shows an obvious gap at around 4 kHz, which has also been shown by previous recordings from the guinea pig auditory cortex^12^. We assume that this gap is based on an adaptive response (either plastic change or inborn characteristic) to the resonance frequency of the bulla, which is at around 4 kHz^35^. This frequency would dominate the auditory percept, if it was not dampened or filtered out. This process is most probably established from the cochlea onwards, as an underrepresentation of CFs around 4 kHz is already apparent in auditory nerve recordings^36^. A comparison of the ECoG grid tonotopy to one derived by PEAs was not possible in the present study, as we did only use one penetration per surface recording position to prevent severe brain damage due to extensive sampling. Besides of the reported similarity to the tonotopic organization in guinea pig A1 derived by different recording methods (see above), we furthermore compare the Q20-values of the surface and penetrating electrode and found no significant difference in sharpness of tuning.

A detailed analysis of the source and spread of the LFP recordings was not within the scope of the study. We did however assess the correlation between ECoG and PEA recordings. The results showed that the signal at the surface was most similar to the ones in superficial layers and the similarity exponentially decreased with cortical depths. This, together with the high (spatial) frequency selectivity, indicates that the LFP recordings in our study are quite local. Similar changes in the LFP signal over only few 100 µm have been reported in the literature^29^. The actual spread may however be less specific for the type of recording electrode and be rather a characteristic of the recorded brain structure and sensory modality^13^.

MUA at the ECoG contacts was similar to the one recorded at the PEA, with similar psth response profiles and input-output characteristics. When comparing MUA to LFP recordings, MUA yielded higher response thresholds, lower dynamic ranges and higher response-background ratios (based on MUA rate)^26^. The tonotopic slope was similar to the one derived from LFP measures. However, the Q20-values were slightly higher (sharper tunings) for the MUA as compared to the LFP recordings. All these differences were not specific for the surface ECoG grid, but were also obvious at the PEA, confirming the general difference between MUA and LFP recordings. Most of the results are in line with the notion that MUA is spatially more selective, integrating activity over a smaller sample of neurons, than the LFP recordings^8^. The higher spatial selectivity will additionally be sharpened by lower volume conduction for MUA as compared to LFP, with less overlap between sources^12^ and by differences in the characteristics of the sources^13^. As the LFP is dominated by the cortical input, whereas the MUA corresponds to the output of the respective neurons, lower frequency selectivity in sub-cortical as compared to cortical structures will aid to the observed difference in frequency selectivity^37^. The elevated response thresholds for MUA (see also Norena & Eggermont, 2002)^37^ can also be interpreted this way, considering that at response threshold the synchrony of activation is important for detection and that variance in a small set of neurons (MUA) will lead to higher uncertainty than the same variance in a larger set of neurons (LFP). Additionally, LFPs may be influenced by cortical input that is too small in amplitude to generate local MUA^37^. The higher response-background ratio at 20 dB above response threshold is likely to be based on differences in background activity for LFP amplitudes as compared to MUA rates. LFP recordings, integrating over a larger population of neurons, will result in a higher background activity than MUA that is based on a smaller set of neurons. However, due to the difference in the stimulus characteristics (field vs. spikes), a direct comparison between the levels of background activity was not possible.

The fact that we revealed very similar slopes for CF-changes along the tonotopic axis for both LFP and MUA measures seemed at first sight to be contradictory to the assumption of a higher spatial acuity for MUA recordings. For example, Fallon and colleagues^17^ reported that they needed to calculate the second spatial derivative (SSD) to reveal similar tonotopic slopes of surface LFP recordings compared to MUA PEA recordings. However, for the visual cortex (V1) of macaques, Xing and colleagues^38^ found similar visual field maps for simultaneously recorded LFP and MUA at the same penetrating electrode and CF values have typically been reported to be similar between LFP and MUA PEA recordings^12,37^. We assume that the contact size and distance of the (surface or penetrating) recording electrodes to the respective neurons, in relation to their functional selectivity (e.g. frequency tuning), is determining whether or not the higher spatial acuity of MUA recordings will result in significant differences of local characteristics (e.g. CF), as compared to LFP recordings.

To assess the link between ECoG and PEA MUA we first compared the FSL and revealed similar FSL between the ECoG grid recordings and recordings from supragranular layers. Furthermore, the MUA trains at the surface were best correlated with trains recorded at the granular layer IV (800–1100 µm)^28^. As the granular layer showed higher spiking activity than supragranular layers (Fig. 5A), this may be an artifact of the correlation computation, as lower correlation coefficients may result from less variability in the data (i.e. few spikes)^39^. However, adding the signals of all 16 contacts did not result in further significant improvement of the correlation. Thus, we conclude that the MUA recorded at the ECoG grid corresponds to supragranular and granular (~900 µm depth) spiking activity. It is however unlikely that an individual granular spike is picked up at a subdural ECoG contact, as the horizontal spread of MUA is supposed to be around 200 µm^6,15,40,41^. We assume that the MUA (or hash) recorded at the surface corresponds to a summation of simultaneous spiking activity^40^ of layer 2/3 pyramidal cells, which may also include activity from layer 1 interneurons^15^ and backwards propagating, dendritic spikes from deeper layers^42^. Further research, such as intracortical electrical stimulation^43^ and pharmacological blockage, will be necessary to characterize the origin of subdural spiking activity recorded with the ECoG grid.

The fact that the newly developed surface electrode also enabled recordings of MUA was quite unexpected, as previous publications usually do not document this feature (for review see Im & Seo, 2016)^44^, or link it to specific properties of the electrode, such as organic interface materials^15^. However, gold and platinum ECoGs have already been described to be able to record MUA from basal root ganglia in the cat, but not in humans, and the authors^20^ conclude that the distance between electrode and tissue might be the key issue for recording unit-activity and suggest that reduced impedances and flexible substrate material would improve the recording quality. The ECoG grid in our study addressed both and provided a flexible and adhesive substrate with low impedance contacts. Due to the lack of systematic analysis of the potential to record surface MUA in current publications (probably related to a reluctance to report negative results), we cannot estimate which material characteristics of the ECoG are critical for this feature. The MUA recording yields several advantages as compared to sole LFP recordings. In addition to the higher spatial acuity, the MUA is also known to allow assessing temporal aspects with higher fidelity. For example, phase locking to repetitions within the signal above 16 Hz is possible, which is the limit for LFP frequency following responses^45^. By this, the MUA is able to reflect for example the fundamental frequency of speech^46^.

## Conclusion

Taken together, the study revealed that the new ECoG grid is a good alternative to conventional PEA considering the comparable signal quality and the potential to record LFP as well as spatially selective MUA. Additionally, they provide the advantage of faster sampling from multiple cortical sites simultaneously, without the risk of tissue damage. The recordings can easily be combined with PEA recordings, whenever layer specific information is needed. The next step would be biocompatibility testing during chronic preparations. Based on the intimate contact to the brain tissue and the stability over several recording sessions, we expect the ECoG grid to be a relevant tool for chronic recordings in the behaving animal.

### Dataset availability

The datasets generated during and/or analyzed during the current study are available from the corresponding author on reasonable request.

## Electronic supplementary material

Supplement Figure 1
