# Supplementary material for: New thin-film surface electrode array enables brain mapping with high spatial acuity in rodents
Source: Sci Rep. 2018 Feb 28;8:3825. doi: 10.1038/s41598-018-22051-z (PMC5830616; doi:10.1038/s41598-018-22051-z)
Supplement: Supplementary file 1 — Supplement Figure 1 [file 41598_2018_22051_MOESM1_ESM.pdf]

New thin-film surface electrode array enables brain mapping with high spatial acuity in rodents

W.S. Konerding[1], U.P. Froriep[2], A. Kral[1], P. Baumhoff [1]

[1]Institute of AudioNeuroTechnology and Department of Experimental Otology, ENT Clinics, Stadtfelddamm 34, Hannover Medical School, 30625 Hannover, Germany

[2] Translational Biomedical Engineering, Fraunhofer Institute for Toxicology and Experimental Medicine (ITEM), Nikolai-Fuchs-Strasse 1, 30625 Hannover, Germany

Supplement Figure 1

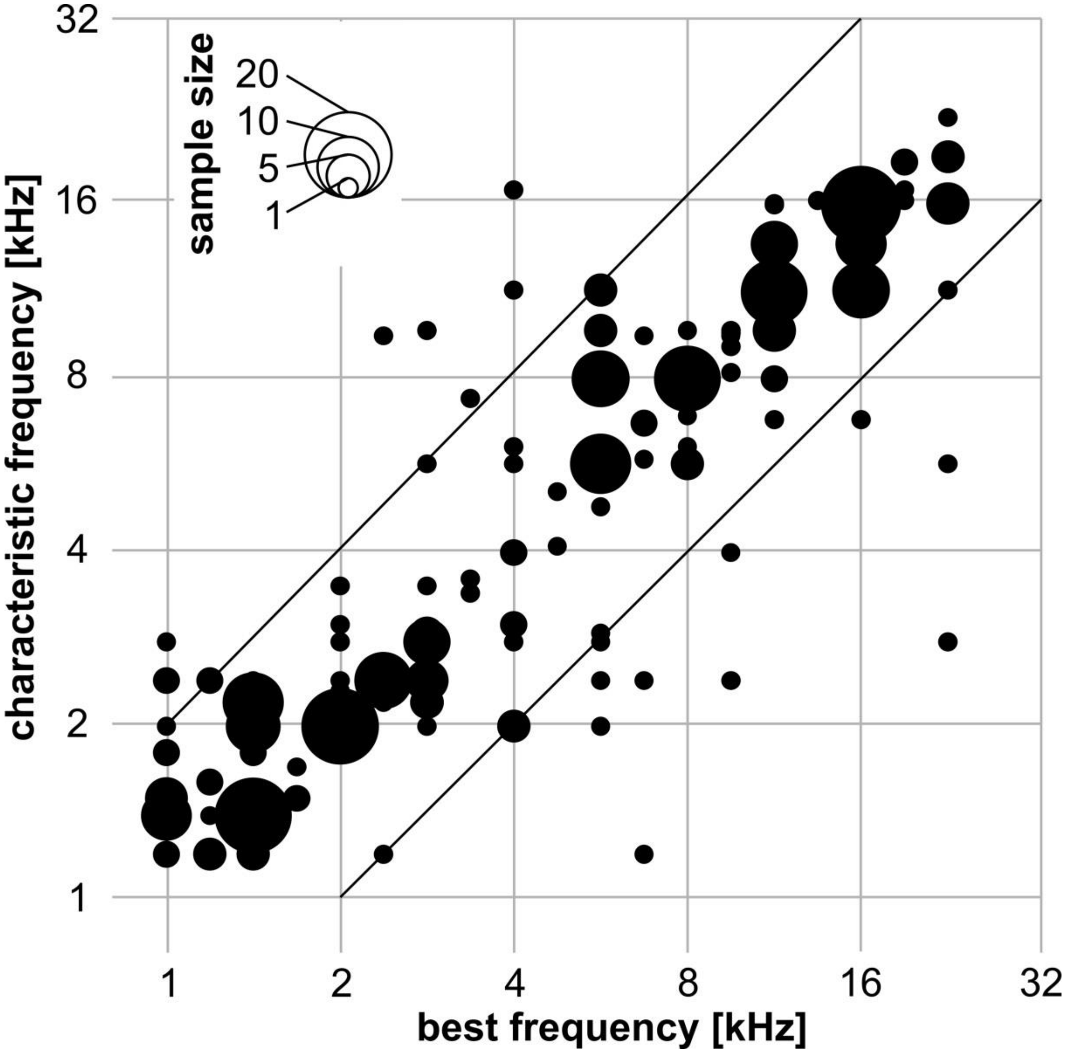

Correlation between best frequency and characteristic frequency

The bubble plot shows best frequency (BF) and characteristic frequency (CF) of each recording position (N=317), whereby the bubble sizes indicates the respective sample size of each BF-CF pair (see legend). The correlation is highly significant (Spearman correlation: $p<0.001$, $r=0.915$) with most data falling within ± 1 octave (solid lines) from the exact match (diagonal).
